# The shift from “MIC-Only” back to carbapenemase testing among carbapenem-resistant Enterobacterales: what clinical laboratories need to know about updated CLSI guidance

**DOI:** 10.1128/jcm.00451-25

**Published:** 2026-03-18

**Authors:** Patricia J. Simner

**Affiliations:** 1 Division of Clinical Microbiology, Department of Laboratory Medicine and Pathology, Mayo Clinic 6915, Rochester, Minnesota, USA; Vanderbilt University Medical Center, Nashville, Tennessee, USA

**Keywords:** carbapenemase, carbapenem-resistant Enterobacterales, CRE, CLSI

## Abstract

Carbapenem-resistant organisms (CROs) pose a major threat to global health due to limited therapeutic options and their capacity for rapid dissemination. Among these, carbapenemase-producing (CP) strains are of greatest concern, as they hydrolyze most β-lactams, and carbapenemase genes are readily spread via mobile genetic elements. Historically, clinical laboratories relied solely on minimal inhibitory concentration (MIC) results and interpretive criteria to guide therapy, with carbapenemase testing performed mainly for epidemiologic purposes. However, changing carbapenemase epidemiology, the introduction of enzyme-specific novel β-lactam combination agents for treatment, and updated Clinical and Laboratory Standards Institute (CLSI) guidance have renewed the importance of carbapenemase testing among carbapenem-resistant Enterobacterales. The 2025 CLSI M100 update now recommends carbapenemase testing for most Enterobacterales resistant to at least one carbapenem, emphasizing differentiation of key enzymes, such as KPC, NDM, and OXA-48-like, to inform therapeutic decisions and support antimicrobial stewardship. This minireview summarizes the evolution of CLSI guidance from early breakpoint establishment through the “MIC-only” era to the current antimicrobial resistance mechanism-driven framework. Key issues addressed include the clinical limitations of prior clinical breakpoints, challenges in balancing sensitivity and specificity of screening criteria to guide carbapenemase testing in different settings, and the expanding role of rapid phenotypic and molecular detection methods. Revisions to the EDTA-modified carbapenem inactivation method (eCIM) are discussed in light of increasing co-production of metallo-beta-lactamase and serine-carbapenemases. Reintegration of carbapenemase testing into clinical workflows highlights the role of the clinical microbiology laboratory as a critical component of antimicrobial stewardship.

## INTRODUCTION

Carbapenem-resistant organisms (CROs) are a critical public health concern worldwide due to increasing prevalence and limited therapeutic options (1, 2). This group includes all gram-negative organisms resistant to any carbapenem tested, as appropriate for the specific organism (e.g., ertapenem is not evaluated for the glucose-non-fermenters as they are intrinsically resistant). Within this group, carbapenemase-producing (CP) carbapenem-resistant organisms (CP-CROs) are especially worrisome as carbapenemases inactivate most β-lactams and spread efficiently via mobile genetic elements (e.g., plasmids) driving carbapenem resistance among Gram-negative organisms. CP-CRO, including Enterobacterales, *Pseudomonas aeruginosa*, and *Acinetobacter baumannii* complex, pose the greatest therapeutic and epidemiological challenge (3). Over the last decade, many changes have occurred, resulting in the necessity for clinical laboratories to perform carbapenemase testing to inform patient management. These changes include (i) the increasing prevalence and changing epidemiology of carbapenemases, (ii) the availability of novel beta-lactam combination agents with specificity to targeted carbapenemases, (iii) the emphasis on antimicrobial stewardship and preservation of agents to reduce the emergence of antimicrobial resistance, and (iv) the availability of novel diagnostics to more rapidly detect certain antimicrobial resistance genes to guide therapeutic management. The focus of this minireview will be on recent Clinical and Laboratory Standards Institute (CLSI) changes to the guidance for carbapenemase testing among carbapenem-resistant Enterobacterales (CRE).

## MECHANISMS OF CARBAPENEM RESISTANCE

Antimicrobial resistance (AMR) mechanisms mediating carbapenem resistance among Gram-negatives include a combination of mechanisms which encompass porin modification/loss, changes to efflux pump substrate specificity/upregulation, penicillin-binding protein modification and/or β-lactamase production (4). Broadly, CROs are divided into carbapenemase-producing (CP) versus non-carbapenemase-producing (non-CP) due to mechanisms other than carbapenemase production. For CRE, non-CP-CRE are mediated most commonly due to cell wall permeability defects (e.g., porin mutations/upregulation of efflux pumps) in combination with non-carbapenem hydrolyzing beta-lactamase production, such as extended-spectrum β-lactamase (ESBL) and/or AmpC β-lactamase (AmpC) production (5).

CP-CROs are the most clinically significant due to their mobility and broad spectrum driving increasing carbapenem resistance rates globally. Furthermore, CP-CRO infections are associated with a higher risk of morbidity and mortality as compared with non-CP-CRO (6, 7). Carbapenemases are enzymes that are capable of hydrolyzing most conventional beta-lactam agents (e.g., penicillins, monobactams, narrow- and expanded-spectrum cephalosporins, and carbapenems). They are classified based on their active site into serine with an active site serine and metallo-beta-lactamases that require at least one zinc ion at their active site. They are further classified based on their amino acid homology into Ambler groups A through D (8). The most common enzyme types are (i) *Klebsiella pneumoniae* carbapenemase (KPC, Ambler class A), (ii) metallo-beta-lactamases (MBLs), including New Delhi metallo- β-lactamase (NDM), Verona integron metallo-β-lactamase (VIM), imipenemase (IMP, Ambler class B), and (iii) oxacillinase (OXA)-48-like among *Enterobacterales* or OXA-23/24/58 variants among *Acinetobacter* species (Ambler class D) (3). Emerging evidence shows that co-occurrence of two or three carbapenemases is increasingly common among CRE, including the combinations of serine and MBL production (e.g., NDM and OXA-48-like) (9, 10).

Carbapenem resistance mechanism testing has focused on detecting CP from non-CP CRO. It should be noted that methods to further define non-CP mediated mechanisms of carbapenem resistance (e.g., efflux, porins, in combination with non-carbapenemases beta-lactamases, etc.) are generally relegated to research settings where whole genome sequencing is required to identify multiple contributing mechanisms (e.g., AmpC beta-lactamase gene and porin mutations). Non-carbapenem hydrolyzing beta-lactamases are often co-produced with carbapenemases, further complicating expected beta-lactam susceptibility patterns and interpretation of results. For example, co-production of ESBL and/or AmpC beta-lactamases among MBL producers will result in aztreonam resistance despite aztreonam not being hydrolyzed by MBL enzymes.

## EVOLUTION OF GUIDANCE ON CARBAPENEMASE TESTING

**Fig 1:**
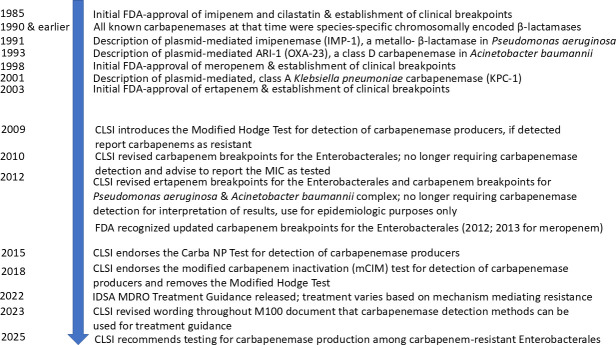
Timeline of changes to carbapenem breakpoints and carbapenemase testing guidance from CLSI over the years.

The guidance and necessity of detecting carbapenem resistance mechanisms has changed over the years, as described below. Fig. 1 summarizes the major changes since the first carbapenem breakpoints for imipenem were established in 1985 to the most recent guidance from CLSI in 2025 recommending carbapenemase testing among CRE.

### Early era—high clinical breakpoints and no carbapenemase testing

When carbapenem breakpoints were first established, carbapenem resistance was rare and plasmid-mediated carbapenemase genes were virtually unknown. Breakpoints were therefore set relatively high (e.g., meropenem ≤4 µg/mL for susceptibility among Enterobacterales), and mechanism-based testing was not necessary. However, with the emergence of plasmid-mediated carbapenemases in the 2000s, these interpretive criteria proved problematic as isolates harboring carbapenemases often tested susceptible, leading to missed detection and clinical failures (11).

To address this, the Modified Hodge Test (MHT) was introduced by CLSI in 2009 as a phenotypic assay for detecting carbapenemase production among Enterobacterales isolates with elevated carbapenem minimal inhibitory concentrations (MICs; e.g., 2 to 4 µg/mL for meropenem). CLSI recommended that any isolate testing positive by MHT should have all carbapenems reported as resistant, regardless of MIC. This was largely driven by the need to identify KPC-producing CRE, which were rapidly spreading in the United States and had few effective treatment options at the time.

### 2010–2012*:* breakpoint revisions and removal of routine MHT

From 2010 to 2012, CLSI lowered carbapenem breakpoints (e.g., meropenem ≤1 µg/mL for susceptibility among Enterobacterales), reflecting pharmacokinetic/pharmacodynamic and clinical outcome data. The rationale was that with these more stringent cutoffs, carbapenemase producers would generally no longer test susceptible. Once laboratories started using the updated clinical breakpoints, the recommendation was to report the MIC as tested regardless of whether it was a carbapenemase producer or not. Treatment recommendations for infections caused by Enterobactearles not susceptible to carbapenems (e.g., meropenem MICs > 1 µg/mL) at that time focused on the carbapenem MIC to guide potential extended-infusion carbapenem use in combination with another active agent from a different antimicrobial class (e.g., fluoroquinolone, aminoglycoside, or polymyxin) (7). Consequently, CLSI removed the requirement for carbapenemase testing, shifting carbapenemase testing toward epidemiologic and infection prevention and control (IP&C) purposes rather than therapeutic guidance. From an IP&C standpoint, detection within the hospital setting is important to guide containment strategies, such as contact precautions and/or patient cohorting, to reduce the spread, especially among high-risk patients. The FDA then subsequently recognized all the current CLSI carbapenem breakpoints (12).

### 2010s–2020: the “MIC-only era” and its limitations

For much of the following decade, laboratories relied on carbapenem MIC and associated interpretive criteria to guide therapy. Carbapenemase detection was primarily performed in reference or public health laboratories. However, the approval of novel β-lactam combination agents fundamentally changed this landscape. Because the niche activity of these agents (e.g., ceftazidime-avibactam for KPC and some OXA-48-like; aztreonam-avibactam or cefiderocol for MBLs), carbapenemase testing became increasingly relevant to patient care (13). Furthermore, the prevalence of CP-CRO continued to increase globally during this time. As such, additional carbapenemase testing methods were developed and implemented in clinical microbiology laboratories to help guide patient care, including for therapeutic guidance (see carbapenemase testing methods section below for further details).

### 2022 onward: IDSA/ESCMID treatment guidance and the rise of mechanism-driven therapy

Beginning in 2022, the Infectious Diseases Society of America (IDSA) and the European Society of Clinical Microbiology and Infectious Diseases (ESCMID) published AMR treatment guidance and provided structured therapeutic recommendations for resistant Gram-negatives, including ESBL-, AmpC-, and carbapenemase-producing Enterobacterales, difficult-to-treat *P. aeruginosa*, carbapenem-resistant *A. baumannii* complex, and *Stenotrophomonas maltophilia* (13, 14). Based on the accumulation of evidence, these guidelines explicitly encourage carbapenemase testing in clinical laboratories to inform therapy of carbapenemase producers. Table 1 summarizes preferred treatment based on carbapenemase genotype among CRE. Regardless of the mechanism mediating resistance, phenotypic AST should always be performed to inform therapy decisions as the activity of novel agents is not guaranteed, and resistance has emerged even to the novel beta-lactam combination agents (15).

**TABLE 1:** Preferred treatment options and laboratory testing and reporting guidance based on the carbapenemase genotype among CRE^*a*^

Carbapenemase genotype	Preferred treatment guidance	Laboratory testing and reporting guidance
KPC	Ceftazidime-avibactam, meropenem-vaborbactam, or imipenem-relebactam	If cefepime S/SDD, suppress or force results to resistant If KPC detected, setup AST or cascade report preferred treatment options based on local formulary
Metallo-beta-lactamases (NDM, VIM, IMP) Or dual serine and MBL producers (e.g., NDM and OXA- 48-like)	Cefiderocol, aztreonam-avibactam (or the combination aztreonam and ceftazidime-avibactam)	If detected, setup or cascade report preferred treatment options based on local formulary
OXA-48-like	Ceftazidime-avibactam	If detected, setup or cascade report preferred treatment options based on local formulary If meropenem-vaborbactam S, suppress or force results to resistant
Any carbapenemase without further differentiation	Empiric therapy selected based on local epidemiology, severity of infection and patient risk factors	If cefepime S/SDD, suppress or force results to resistant If detected, set up or cascade report preferred treatment options based on local formulary. Recommend including one agent active against MBLs

^*a*^ KPC: *Klebsiella pneumoniae* carbapenemase; NDM: New Dehli metallo- β-lactamase, VIM: Verona integron metallo- β-lactamase, IMP: imipenemase, OXA-48-like: Oxacillinase (OXA)-48- like; S: susceptible, SDD: susceptible-dose dependent.

### 2019–2023: reporting challenges and CLSI revisions

Carbapenemase testing also impacts the clinical microbiology laboratory and reporting practices. In 2019, data presented at a CLSI meeting showed that 14%–23% of KPC-producing Enterobacterales tested susceptible (S) or susceptible-dose dependent (SDD) to cefepime. Initially, CLSI continued to allow cefepime reporting as tested among CP-CRE with cautionary comments about the use of cefepime when S/SDD due to lack of outcomes data (16, 17).

Subsequent murine thigh model studies demonstrated that cefepime failed to achieve ≥1 log bacterial kill against CP-CRE, even when isolates tested susceptible, especially compared with non-CP-CRE. This evidence, presented to CLSI in 2023, led to the recommendation that cefepime S/SDD results be suppressed or reported as resistant for CP-producers to discourage use in these cases (16, 18). A recent case report by Morelan et al. illustrates how delayed recognition of a KPC-producing *E. coli* urinary tract infection resulted in suboptimal cefepime therapy, despite *in vitro* susceptibility (19). Timely detection of KPC, coupled with updated guidance to suppress cefepime susceptibility results in the presence of carbapenemases and other reporting strategies, could have facilitated the earlier administration of a more appropriate antimicrobial therapy and prevented the need for readmission in this case (20). Further highlighting the role of the clinical microbiology laboratory in antimicrobial stewardship.

Another reporting challenge involves meropenem-vaborbactam in OXA-48-like producers. Although breakpoints are higher than meropenem alone due to optimized dosing (2 g q8h extended infusion), vaborbactam lacks OXA-48-like inhibitory activity. Murine models showed failure to achieve ≥1 log killing in OXA-48-like producers despite susceptible meropenem-vaborbactam MICs, prompting CLSI to recommend suppression or resistant reporting in these cases as well (16, 21). Thus, carbapenemase testing is increasingly important for both laboratory reporting practices and to help guide therapeutic management (Table 1). To begin to address this, CLSI revised wording throughout the M100 document in 2023 to endorse carbapenemase testing for therapeutic guidance, in addition to guiding epidemiologic and IPC initiatives. However, there were no direct recommendations at that time for laboratories to perform carbapenemase testing.

## THE CURRENT ERA - RECENT CLSI UPDATES TO CARBAPENEMASE TESTING RECOMMENDATIONS

In 2025, CLSI reinstated recommendations that all Enterobacterales resistant to at least one carbapenem tested (ertapenem, meropenem, or imipenem) undergo carbapenemase testing in addition to standard phenotypic antimicrobial susceptibility testing (AST). This is framed as a recommendation, not a requirement. Importantly, the guideline provides exceptions for species with intrinsic resistance mechanisms, such as *Proteus*, *Providencia*, and *Morganella* spp. resistant only to imipenem, since their elevated imipenem MICs are typically mediated by non-carbapenemase mechanisms. CLSI emphasizes that carbapenemase testing should aim not only to detect carbapenemase activity but also, ideally, to differentiate the specific carbapenemase genotype (e.g., KPC, NDM, OXA-48-like) to help with therapeutic guidance. Despite reinstating carbapenemase testing, CLSI does not recommend modifying the interpretation of the carbapenems even if one or multiple of the carbapenems test susceptible among carbapenemase-producers. Appendix G in the M100 provides guidance on discrepancies between carbapenemase detection and carbapenem susceptibility testing results. Laboratories may consider additional confirmatory testing or reporting the results as tested along with a comment advising caution as current clinical and laboratory evidence is insufficient to conclude whether carbapenem monotherapy of carbapenemase producers with an MIC in the susceptible range will be effective (16).

### Balancing sensitivity and specificity in screening

Screening criteria to guide carbapenemase testing will vary by institution, depending on local epidemiology and available resources. The choice of criteria requires balancing sensitivity (avoiding missed carbapenemase producers) and specificity (avoiding unnecessary testing, cost, and labor). Ertapenem is the most sensitive carbapenem for detecting carbapenemase production in Enterobacterales. Data presented at the January 2024 CLSI meeting (SENTRY program; personal communication, Mariana Castanheira) demonstrated that ertapenem not susceptible results (intermediate or resistant) detected nearly all major carbapenemases: KPC: 98.6%, NDM: 99.5%, OXA-48-like: 97.1%, IMP: 95.5%, and overall sensitivity for the “big five” carbapenemases: 97.0%. In contrast, VIM producers were more reliably detected when using imipenem not susceptible results as the screening criterion (22). Thus, local epidemiology should be used to guide screening criteria for carbapenemase testing among CRE.

### Specificity and mono-ertapenem resistance

While ertapenem is highly sensitive, specificity is low for detection of CP-CRE. A mono-ertapenem resistant screening approach (i.e. remains susceptible to other carbapenems tested) had only ~10%–20% specificity across U.S. data sets. Approximately half of CRE isolates encountered clinically in the United States are mono-ertapenem resistant; of those, the likelihood of carbapenemase production varies by species: *Klebsiella pneumoniae*: ~20%*, Escherichia coli*: ~15%*, Enterobacter cloacae* complex: ~5%*,* and *Klebsiella aerogenes*: ~2% (22).

Based on these data, CLSI suggests a possible exception for screening *E. cloacae* complex and *K. aerogenes* isolates resistant to ertapenem only, since carbapenemase production is uncommon and resistance is typically due to alternative mechanisms (e.g., high-level AmpC production in combination with porin loss) in U.S. isolates. Outside the United States, however, the prevalence of carbapenemase production among mono-ertapenem–resistant CRE is significantly higher, and institutions must adapt screening practices accordingly (5).

## CARBAPENEMASE DETECTION METHODS AND ALGORITHMS

**Fig 2:**
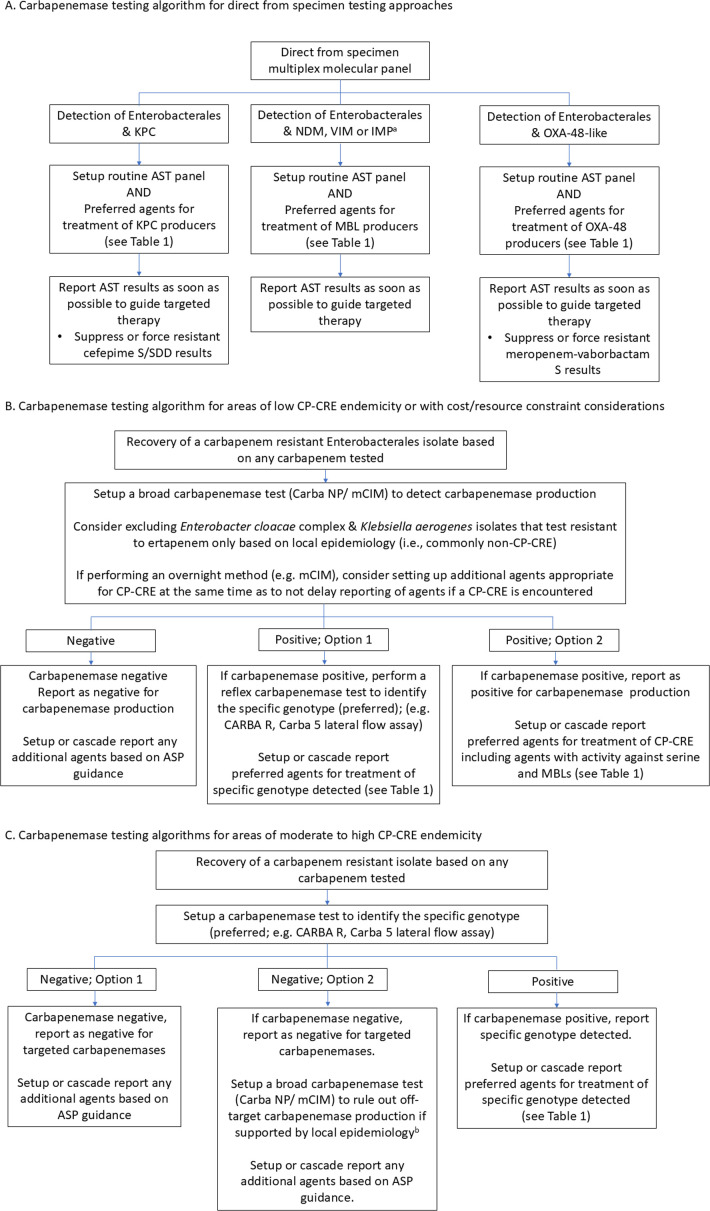
Carbapenemase testing algorithms directly from specimens (**A**) or isolate testing for low (**B**) to high (**C**) carbapenemase-producing CRE prevalence settings. ^a^ A similar testing and reporting algorithm should be considered for dual serine and MBL producers. ^b^ Option #2 may be pursued if alternative carbapenemase targets are commonly encountered, for instance, *bla*_SPM_ in South America or alternative OXA carbapenemases are more common among *Acinetobacter* species, such as *bla*_OXA-23_ or *bla*_OXA-24_.

Methods for detecting and differentiating carbapenemases are described in CLSI M100 and in other reviews (5, 16). Of note, in 2018, CLSI removed the MHT as a CLSI-endorsed test due to the availability of better performing tests (e.g., Carba NP, modified carbapenem inactivation methods [mCIM]). Carbapenemase tests can be divided into broad carbapenemase tests versus carbapenemase differentiating tests. Broad carbapenemase tests detect the production of any carbapenemase, like the Carba NP or mCIM. These methods are able to differentiate carbapenem resistance mediated by carbapenemase production versus non-carbapenemase mediated mechanisms. The specific carbapenemases can be detected and differentiated by molecular (gene-based) or lateral flow assay (LFA) tests (enzyme-based), but these approaches are often limited to certain targets (often VIM, NDM, KPC, IMP, and OXA-48-like). Combined testing algorithms may involve initial screening for carbapenemase production (e.g., mCIM), followed by a carbapenemase differentiation test (e.g., LFA), but algorithms may be institution specific and vary based on prevalence and patient population. Multiple algorithms for carbapenemase testing are outlined in Fig. 2A when direct from specimen molecular testing is performed versus Fig. 2B and C when considering isolate testing in low vs moderate to high CP-CRE endemicity, respectively.

It should be noted that there are limitations to carbapenemase testing methods, including the inability to detect multiple contributing mechanisms mediating resistance. Targeted methods are often limited to the most common carbapenemases and do not detect all possible carbapenemases or even all known allelic variants, even if a target for the assay (e.g., *bla*_IMP_ detection) is often limited to *bla*_IMP-1_ group and does not detect variants outside of the *bla*_IMP-1_ group. Furthermore, genotype to phenotype discrepancies may arise when both testing modalities are applied to detect carbapenemases. These discrepant results should be investigated further. The readers are guided to CLSI M100 Appendix G and to other reviews and publications on how to resolve genotype to phenotype discrepancies (16, 23).

## CHANGES TO eCIM REPORTING DUE TO CHANGING EPIDEMIOLOGY OF CP-CRE

Due to the increasing prevalence of NDM-producing Enterobacterales in the United States and globally and the occurrence of isolates co-producing both MBLs and serine carbapenemases**,** modifications were made to reporting EDTA-mCIM (eCIM) method (9, 10). The eCIM differentiates MBLs from serine carbapenemases through the addition of EDTA, a divalent cation chelator that inhibits MBL activity due to requirement of zinc at the active site for activity.

The eCIM is performed in parallel with the mCIM, but it is only interpreted when the mCIM is positive**,** indicating carbapenemase production. Importantly, the CLSI-endorsed eCIM method is currently restricted to use with Enterobacterales due to performance issues with *P. aeruginosa* during the multicenter CLSI study (24). Other modifications to the eCIM method have been described for use with *P. aeruginosa* (25). A key limitation is observed when an isolate co-produces an MBL and a serine carbapenemase. In this scenario, the activity of the serine carbapenemase can mask the presence of the MBL, as EDTA does not inhibit serine enzymes. This may yield an inconclusive or negative eCIM result**,** even when an MBL is present. To address this, CLSI now recommends that negative eCIM results be interpreted as inconclusive, and that an alternative method be used to rule out MBL production. This limitation reflects a broader challenge inherent to inhibitor-based assays**,** where overlapping or masked enzyme activity may complicate interpretation. Carbapenemase differentiation assays, such as the Carba 5 or CARBA R, are capable of picking up the most common dual producers, such as NDM plus OXA-48-like. However, more specialized methods, such as broader targeted beta-lactamase molecular panels or whole genome sequencing, would be required to detect less frequently encountered or off-target carbapenemases among multiple carbapenemase producers.

## BEYOND CRE

The recent CLSI changes focused on carbapenemase testing recommendations in CRE. However, CP-*P. aeruginosa* and CP-*A. baumannii* complex are a critical concern globally as well and offer different challenges for detection of carbapenemases. For instance, the prevalence of CP among CR-*P. aeruginosa* varies significantly from 2% in the USA to almost 70% in South America. Furthermore, the epidemiology of carbapenemases harbored by CP-CR-*P. aeruginosa* varies significantly geographically, where *bla*_VIM_ is the most common in the USA, *bla*_KPC_ in South America and China, and *bla*_IMP_ in Australia and Singapore (6). For *Acinetobacter baumannii* complex, the most common carbapenemases are *bla*_OXA_ variants (non-*bla*_OXA-48_) which are not usually included as targets on carbapenemase differentiation assays. Further, the dual production of NDM plus OXA-type carbapenem hydrolyzing enzymes is important as sulbactam-durlobactam becomes an important agent for treatment of carbapenem-resistant *Acinetobacter baumannii* complex with activity against carbapenem hydrolyzing OXA enzymes but lacking activity against MBL producers. Further complicating detection of CP-CRAB is that many of the CLSI-endorsed methods do not perform as well for this organism and are not recommended for use as described and may need further modifications to enhance performance (e.g., the CLSI Carba NP and mCIM methods are not recommended for use with *A. baumannii* complex isolates) (26, 27). Future updates to CLSI will address CP detection and testing in these organisms. Currently, CLSI is working on defining guidance for screening algorithms and further assessing the methods for detection of CP-CR-*P. aeruginosa*. The updated guidance is expected in the forthcoming M100-S37 document. The readers are guided to a recent review on potential screening and carbapenemase testing algorithms among these organisms for further guidance (5).

## SUMMARY

Carbapenem-resistant organisms (CROs) are an urgent global health threat due to limited treatment options and their ability to spread resistance rapidly. Over the past two decades, shifts in epidemiology, the development of novel β-lactam combination agents, and antimicrobial stewardship priorities have made carbapenemase testing increasingly critical for guiding therapy. Current guidance from CLSI (16) now recommends carbapenemase testing for all Enterobacterales resistant to at least one carbapenem, with emphasis on differentiating specific carbapenemase genotypes (e.g., KPC, NDM, OXA-48-like) to optimize treatment decisions. While ertapenem resistance serves as a sensitive screening tool, species-specific exceptions and low specificity highlight the need for careful interpretation and tailored algorithms. Looking forward, broader application of these principles to CP-*Pseudomonas* and CP-*Acinetobacter* is needed to address the full scope of the antimicrobial resistance crisis.

## References

[1] CDC. 2019. Antibiotic resistance threats in the United States, 2019. Altanta, GA Services DoHaH, CDC. https://www.cdc.gov/antimicrobial-resistance/media/pdfs/2019-ar-threats-report-508.pdf.

[2] Organization WH. 2025. Global antibiotic resistance surveillance report 2025. GLASS GARaUSS. https://www.who.int/publications/i/item/9789240116337.

[3] Hansen GT. 2021. Continuous evolution: perspective on the epidemiology of carbapenemase resistance among Enterobacterales and other gram-negative bacteria. Infect Dis Ther 10:75–92. doi:10.1007/s40121-020-00395-2 33492641 PMC7954928

[4] Garcia-Bustos V, Cabañero-Navalón MD, Salavert Lletí M. 2022. Resistance to beta-lactams in Gram-negative bacilli: relevance and potential therapeutic alternatives. Rev Esp Quimioter 35 Suppl 2:1–15. doi:10.37201/req/s02.01.2022 PMC9632057 36193979

[5] Simner PJ, Pitout JDD, Dingle TC. 2024. Laboratory detection of carbapenemases among Gram-negative organisms. Clin Microbiol Rev 37:e0005422. doi:10.1128/cmr.00054-22 39545731 PMC11629623

[6] Reyes J, Komarow L, Chen L, Ge L, Hanson BM, Cober E, Herc E, Alenazi T, Kaye KS, Garcia-Diaz J, et al.. 2023. Global epidemiology and clinical outcomes of carbapenem-resistant Pseudomonas aeruginosa and associated carbapenemases (POP): a prospective cohort study. Lancet Microbe 4:e159–e170. doi:10.1016/S2666-5247(22)00329-9 36774938 PMC10016089

[7] Tamma PD, Goodman KE, Harris AD, Tekle T, Roberts A, Taiwo A, Simner PJ. 2017. Comparing the outcomes of patients with carbapenemase-producing and non-carbapenemase-producing carbapenem-resistant enterobacteriaceae bacteremia. Clin Infect Dis 64:257–264. doi:10.1093/cid/ciw741 28013264 PMC5241781

[8] Hall BG, Barlow M. 2005. Revised ambler classification of beta-lactamases. J Antimicrob Chemother 55:1050–1051. doi:10.1093/jac/dki130 15872044

[9] Rankin DA, Stahl A, Sabour S, Khan MA, Armstrong T, Huang JY, Baggs J, Walters MS. 2025. Changes in carbapenemase-producing carbapenem-resistant Enterobacterales, 2019 to 2023. Ann Intern Med 178:1818–1821. doi:10.7326/ANNALS-25-02404 40982973 PMC12645407

[10] Yuan PB, Dai LT, Zhang QK, Zhong YX, Liu WT, Yang L, Chen DQ. 2024. Global emergence of double and multi-carbapenemase producing organisms: epidemiology, clinical significance, and evolutionary benefits on antimicrobial resistance and virulence. Microbiol Spectr 12:e0000824. doi:10.1128/spectrum.00008-24 38860788 PMC11218513

[11] Hong T, Moland ES, Abdalhamid B, Hanson ND, Wang J, Sloan C, Fabian D, Farajallah A, Levine J, Thomson KS. 2005. Escherichia coli: development of carbapenem resistance during therapy. Clin Infect Dis 40:e84–6. doi:10.1086/429822 15844056

[12] FDA. 2024. FDA-recognized antimicrobial susceptibility test interpretive criteria. https://www.fda.gov/drugs/development-resources/fda-recognized-antimicrobial-susceptibility-test-interpretive-criteria.

[13] Tamma PD, Heil EL, Justo JA, Mathers AJ, Satlin MJ, Bonomo RA. 2024. Infectious Diseases Society of America 2024 guidance on the treatment of antimicrobial-resistant gram-negative infections. Clin Infect Dis:ciae403. doi:10.1093/cid/ciae403 39108079

[14] Paul M, Carrara E, Retamar P, Tängdén T, Bitterman R, Bonomo RA, de Waele J, Daikos GL, Akova M, Harbarth S, Pulcini C, Garnacho-Montero J, Seme K, Tumbarello M, Lindemann PC, Gandra S, Yu Y, Bassetti M, Mouton JW, Tacconelli E, Rodríguez-Baño J. 2022. European Society of Clinical Microbiology and Infectious Diseases (ESCMID) guidelines for the treatment of infections caused by multidrug-resistant Gram-negative bacilli (endorsed by European society of intensive care medicine). Clin Microbiol Infect 28:521–547. doi:10.1016/j.cmi.2021.11.025 34923128

[15] Mathers AJ, Peirano G, Pitout JDD. 2015. The role of epidemic resistance plasmids and international high-risk clones in the spread of multidrug-resistant Enterobacteriaceae. Clin Microbiol Rev 28:565–591. doi:10.1128/CMR.00116-14 25926236 PMC4405625

[16] CLSI. 2025. Performance standards for antimicrobial susceptibility testing; thirty-fifth informational supplement. Vol. M100-S35. Wayne, PA CLSI

[17] Fissel JA, Yarbrough ML, Tekle T, Burnham CA, Simner PJ. 2020. Reporting considerations for cefepime-susceptible and -susceptible-dose dependent results for carbapenemase-producing Enterobacterales. J Clin Microbiol 58:e01271-20. doi:10.1128/JCM.01271-20 32641398 PMC7448622

[18] Fouad A, Gill CM, Simner PJ, Nicolau DP, Asempa TE. 2023. Cefepime in vivo activity against carbapenem-resistant Enterobacterales that test as cefepime susceptible or susceptible-dose dependent in vitro: implications for clinical microbiology laboratory and clinicians. J Antimicrob Chemother 78:2242–2253. doi:10.1093/jac/dkad229 37522258

[19] Morelan J, Hou R, Chamberland R, Gill CM. 2025. Cefepime failure for the treatment of cefepime-susceptible, KPC-producing Escherichia coli emphysematous cystitis. ASM Case Rep 1:e00094-25. doi:10.1128/asmcr.00094-25 41244277 PMC12584169

[20] Murphy SG, Simner PJ. 2025. From detection to decision: how laboratory testing and reporting strategies can shape management of carbapenemase-producing Enterobacterales infections. ASM Case Rep 1:e00139-25. doi:10.1128/asmcr.00139-25 41244283 PMC12584174

[21] Anonymous. 2017. VABOMERE (meropenem and vaborbactam) for injection, for intravenous use

[22] CLSI. 2024. 2024 January AST meeting agenda summary minutes

[23] Yee R, Dien Bard J, Simner PJ. 2021. The genotype-to-phenotype dilemma: how should laboratories approach discordant susceptibility results? J Clin Microbiol 59:e00138-20. doi:10.1128/JCM.00138-20 PMC8316082 33441396

[24] Sfeir MM, Hayden JA, Fauntleroy KA, Mazur C, Johnson JK, Simner PJ, Das S, Satlin MJ, Jenkins SG, Westblade LF. 2019. EDTA-Modified carbapenem inactivation method: a phenotypic method for detecting metallo-β-lactamase-producing Enterobacteriaceae. J Clin Microbiol 57:e01757-18. doi:10.1128/JCM.01757-18 30867235 PMC6498035

[25] Gill CM, Lasko MJ, Asempa TE, Nicolau DP. 2020. Evaluation of the EDTA-modified carbapenem inactivation method for detecting metallo-β-lactamase-producing Pseudomonas aeruginosa. J Clin Microbiol 58:e02015-19. doi:10.1128/JCM.02015-19 32238433 PMC7269398

[26] Simner PJ, Johnson JK, Brasso WB, Anderson K, Lonsway DR, Pierce VM, Bobenchik AM, Lockett ZC, Charnot-Katsikas A, Westblade LF, Yoo BB, Jenkins SG, Limbago BM, Das S, Roe-Carpenter DE. 2018. Multicenter evaluation of the modified carbapenem inactivation method and the Carba NP for detection of carbapenemase-producing Pseudomonas aeruginosa and Acinetobacter baumannii. J Clin Microbiol 56:e01369-17. doi:10.1128/JCM.01369-17 29118172 PMC5744225

[27] Uechi K, Tada T, Shimada K, Kuwahara-Arai K, Arakaki M, Tome T, Nakasone I, Maeda S, Kirikae T, Fujita J. 2017. A modified carbapenem inactivation method, CIMTris, for Carbapenemase production in Acinetobacter and Pseudomonas Species. J Clin Microbiol 55:3405–3410. doi:10.1128/JCM.00893-17 28954898 PMC5703807

